# Comparison of 30-day planned and unplanned readmissions in a tertiary teaching hospital in China

**DOI:** 10.1186/s12913-023-09193-1

**Published:** 2023-03-06

**Authors:** Mengjiao Zhang, Siru Liu, Yongdong Bi, Jialin Liu

**Affiliations:** 1 Information Center, West China Hospital, Sichuan University, Chengdu, China; 2 Department of Biomedical Informatics, Vanderbilt University Medical Center, Nashville, TN USA; 3 Department of Medical Informatics, West China Medical School, Sichuan, China

**Keywords:** Patient readmission, Hospital, China

## Abstract

**Purpose:**

The purpose of this study was to analyze and compare the clinical characteristics of patients with 30-day planned and unplanned readmissions and to identify patients at high risk for unplanned readmissions. This will facilitate a better understanding of these readmissions and improve and optimize resource utilization for this patient population.

**Methods:**

A retrospective cohort descriptive study was conducted at the West China Hospital (WCH), Sichuan University from January 1, 2015, to December 31, 2020. Discharged patients (≥ 18 years old) were divided into unplanned readmission and planned readmission groups according to 30-day readmission status. Demographic and related information was collected for each patient. Logistic regression analysis was used to assess the association between unplanned patient characteristics and the risk of readmission.

**Results:**

We identified 1,118,437 patients from 1,242,496 discharged patients, including 74,494 (6.7%) 30-day planned readmissions and 9,895 (0.9%) unplanned readmissions. The most common diseases of planned readmissions were antineoplastic chemotherapy (62,756/177,749; 35.3%), radiotherapy sessions for malignancy (919/8,229; 11.2%), and systemic lupus erythematosus (607/4,620; 13.1%). The most common diseases of unplanned readmissions were antineoplastic chemotherapy (2038/177,747; 1.1%), age-related cataract (1061/21,255; 5.0%), and unspecified disorder of refraction (544/5,134; 10.6%). There were statistically significant differences between planned and unplanned readmissions in terms of patient sex, marital status, age, length of initial stay, the time between discharge, ICU stay, surgery, and health insurance.

**Conclusion:**

Accurate information on 30-day planned and unplanned readmissions facilitates effective planning of healthcare resource allocation. Identifying risk factors for 30-day unplanned readmissions can help develop interventions to reduce readmission rates.

## Introduction

Hospital readmission is a serious, common, and costly adverse patient outcome. Unplanned readmission is not only an indicator of the critical quality of care for patients but also a significant factor in rising healthcare costs [1, 2]. Readmissions account for billions of dollars in annual Medicare expenditures [3]. There is growing recognition that readmission is an outcome measure for quality of healthcare, cost reduction, and transitions of care [4].

Unplanned readmission within 30 days of discharge is an important indicator of the cost and quality of healthcare service and is strongly related to clinical and sociodemographic characteristics [5, 6]. Reducing readmissions is a priority for hospitals and clinicians to improve the quality of healthcare and reduce costs. To address readmissions, the Centers for Medicare and Medicaid Services developed the Hospital Readmissions Reduction Program (HRRP) in 2010 and implemented it in 2012 [7, 8]. Its purpose is to encourage hospitals to improve the quality and transition of healthcare to a better plan of discharge, thereby effectively reducing avoidable 30-day readmissions [8]. The first important step in reducing readmissions is to determine the incidence, risk factors, and causes of readmission. This information can help identify patients at high risk of readmission and target interventions to reduce avoidable readmissions.

Research on the relationship between healthcare quality and readmission needs to distinguish between planned and unplanned readmissions, as only unplanned readmissions can reflect the healthcare quality at the first hospitalization. Planned readmissions may be associated with the utilization of hospital resources (multiple admissions for reimbursement purposes or therapeutic procedures), but not with the healthcare quality process [9]. One study showed that patients with hematological and oncological diseases, renal disease, heart failure, and chronic obstructive pulmonary disease had the highest odds of unplanned readmission across all age groups [10]. However, another study found no risk factors for readmission, except that readmitted patients were significantly older than those who were not readmitted [11]. Despite a large number of readmission studies, it is unclear whether planned and unplanned 30-day readmissions differ between hospitals. The reasons for unplanned readmissions are also not fully elucidated [10, 11]. The relative contribution of patient-level risk factors and structural hospital characteristics to the variation in unplanned readmissions is not fully understood. As reported by the OECD, identifying truly unplanned readmissions is complex [12].

This study aimed to describe the incidence of planned and unplanned 30-day readmissions and to investigate the incidence of time. We sought to analyze the characteristics of readmitted patients and to identify risk factors associated with unplanned readmission. This will provide a basis for improving the quality of healthcare and optimizing the discharge process.

## Method

### Patients and setting

A retrospective descriptive cohort study was conducted at West China Hospital (WCH), Sichuan University. WCH is a 4300-bed teaching hospital in Sichuan, one of the best and largest hospitals in China. In 2021, more than 7.75 million patients visited the outpatient and emergency departments, 279,000 patients were discharged from inpatient departments, and more than 196,000 operations were performed (http://www.wchscu.cn/Home.html). The study cohort includes patients discharged between 1 and 2015 and 31 December 2020. Only patients aged 18 years or older at the time of the index admission were included. All patient data were obtained from the hospital’s electronic health record (EHR).

### Study variables

In this study, we used six years (2015–2020) of discharge EHR with relevant information on patient characteristics and hospital admissions (e.g., date of admission and discharge, principal diagnosis). This included information on patient demographic characteristics (e.g., age, sex, marital status, type of health insurance) and clinical characteristics (e.g., length of stay, surgery or not, ICU stay or not). A patient was readmitted if a new admission occurred within 30 days of the first discharge and was related to the index admission. Day patients and outpatients were excluded. Transfers between units within the same hospital and between hospitals were not considered as readmissions.

The patient ID was used to identify all patients who were readmitted within 30 days. These patients created a 30-day readmission group and a 30-day non-readmission group. Planned versus unplanned readmissions were identified by revisiting the medical records of all patients readmitted within 30 days. Planned readmission was defined as an intentionally planned readmission during the index admission, and patients without a planned readmission were defined as unplanned readmissions. Diagnosis was determined using the International Classification of Diseases, 10th Revision (ICD-10). Patient demographic and clinical characteristics were obtained from the EHR.

The main outcome measure of this research was unplanned and planned 30-day readmission to the hospital. Second, the risk factors for unplanned 30-day readmission were analyzed.

### Statistical analysis

All variables were reported before analysis using frequencies and percentages or means, medians, and standard deviations. The distributions of continuous variables were assessed using histograms. Univariate analysis and bivariate logistic regression analyses were performed for unplanned readmission within 30 days. For univariate analysis, we used the Student t-test for continuous variables and the chi-square test for categorical variables. We performed all statistical analyses using IBM SPSS Statistics 20. Statistical significance was determined by p < 0.05.

### Ethics statement

The study was approved by the Bioethics Committee of the West China Hospital Sichuan University (2022 − 174). Only information that was routinely collected during hospitalization was used. We used anonymous electronic medical records, so we did not seek written consent from participants.

## Results

In January 2015 and December 2020, data on 1,242,496 discharged patients were available for analysis. We excluded 124,059 patients due to death (7,496) and patients aged < 18 years (116,563), leaving a total of 1,118,437 patients for the analysis dataset. Of the 1,118,437 patients, 84,389 were readmitted within 30 days of discharge. This included 74,494 (6.7%) planned readmissions and 9,895 (0.9%) unplanned readmissions. There were significantly more women with planned readmissions than unplanned readmissions, 67.0% and 51.3% respectively. The age groups with the highest number of planned and unplanned readmissions were 40≤-<49 years (21,653; 29.1%) and 50≤-<59 years (2,074; 21.0%). The number of days of initial hospitalization for patients with planned and unplanned readmissions was predominantly in the 1≤-<4-day group, 67.0% (49,936) and 46.5% (4,600), respectively (Table 1). The age group with the lowest number of patients with both planned and unplanned readmissions was ≥ 80 years (0.9% vs. 4.2%). Table 1 shows the demographics and associated risk factors for planned and unplanned readmissions.

Bivariate logistic regression was used to identify variables independently associated with an increased risk of 30-day unplanned readmission. In a bivariate logistic regression model, factors significantly associated with 30-day unplanned readmission were age, LOS, sex (male), marital status (separated/divorced, single, widowed/other), ICU stay, and surgery (Table 2).

**Table 1:** Characteristics of patient readmissions

	Planned readmission n = 74494	Unplanned readmission n = 9895	x^2^/t	p
**Gender**				
female	49,938(67.0%)	5,073(51.3%)	956.98	P<0.001
male	24,556(33.0%)	4,822(48.7%)		
**Marital status**				
Married/partner	68,879(92.4%)	8,236(83.2%)		
Separated/divorced	1,395(1.9%)	224(2.3%)	1,117.38	P<0.001
single	2,959(4.0%)	1,055(10.7%)		
Widowed/other	1,261(1.7%)	380(3.8%)		
Age years				
18≤-<30	3,513(0.5%)	1,243(12.5%)		
30≤-<39	7,631(10.2%)	1,213(12.3%)		
40≤-<49	21,653(29.1%)	1,929(19.5%)		
50≤ -<59	21,346(28.7%)	2,074(21.0%)	2584.87	P<0.001
60≤-<69	15,463(20.8%)	1,941(19.6%)	5	
70≤ -<79	4,213(5.7%)	1,079(10.9%)		
≥ 80	675(0.9%)	416(4.2%)		
Mean(SD)	51.59(12.41)	51.71(16.83)	-0.64	p<0.52
LOS (days)				
1≤-<4	49,936(67.0%)	4,600(46.5%)		
4≤-<7	11,397(15.3%)	1,374(13.9%)		
7≤-<14	9,560(12.8%)	2,173(22.0%)	3382.70	P<0.001
14≤-<20	1,612(2.2%)	737(7.4%)	8	
20≤-<30	1,297(1.7%)	644(6.5%)		
≥ 30	692(0.9%)	367(3.7%)		
Mean(SD)	4.09(6.78)	8.04(12.19)	31.57	P<0.001
IDR (days)				
1 ≤-<3	1,262(1.7%)	1,093(11.0%)		
3 ≤-<5	936(1.3%)	364(3.7%)		
5≤<7	3,485(4.7%)	1,165(11.8%)		
7 ≤ <10	10,159(13.6%)	790(8.0%	4285.42	P<0.001
10≤ <20	16,980(22.8%)	1,964(19.8%)	5	
20≤ <30	41,672(55.9%)	4,519(45.7%)		
Mean(SD)	18.54(7.96)	16.18(10.01)	22.54	P<0.001
No stay in ICU	74,394(99.9%)	9,704(98.1%)	819.92	P<0.001
Stay in ICU	100(0.1%)	191(1.9%)		
Surgery				
No	47,694(64.0%)	4,131(41.7%)	1,828.96	P<0.001
Yes	26,800(36.0%)	5,764(58.3%)		
Health insurance				
Yes	67,765(91.1%)	8,395(84.8%)	372.67	P<0.001
No	2322(3.1%)	525(5.3%)		
Missing	4407(5.8%)	975(9.9%)		

LOS: Length of initial stay; IDR: Interval from discharge to readmission

**Table 2:** Bivariate logistic regression analysis of risk factors for 30-day unplanned readmission

	B	S.E.	P value	Exp(B)	95% C.I.	
Age years	0.001	0.001	0.423	1.001	0.999	1.002
LOS (days)	0.05	<0.001	<0.001	1.05	1.05	1.05
IDR (days)	-0.03	<0.001	<0.001	0.97	0.96	0.97
Gender	0.66	0.02	<0.001	1.93	1.85	2.02
Marital			<0.001			
Separated/divorced	0.30	0.07	<0.001	1.34	1.16	1.55
single	1.09	0.04	<0.001	2.98	2.77	3.21
Widowed/other	0.92	0.06	<0.001	2.52	2.24	2.83
ICU	2.68	0.12	<0.001	14.64	11.49	18.67
Surgery	0.91	0.02	<0.001	2.48	2.38	2.59
Health insurance			<0.001			
No	-0.58	0.04	<0.001	0.56	0.52	0.60
Missing	0.02	0.06	0.72	1.02	0.91	1.15

### Comparison of diseases with planned and unplanned readmissions

The most common disease for planned readmissions was antineoplastic chemotherapy (62,756/177,749; 35.3%), followed by radiotherapy sessions for malignancy (919/8,229; 11.2%), and systemic lupus erythematosus (607/4,620; 13.1%). The most common diseases of unplanned readmission was antineoplastic chemotherapy (2038/177,747; 1.1%), followed by age-related cataract (1061/21,255; 5.0%), and unspecified disorder of refraction (544/5,134; 10.6%) (Table 3).

**Table 3:** Disease readmission numbers and rates

	Planned readmission			Unplanned readmission				
Disease	N	Discharged	(%)	Disease	N	Discharged	(%)	
Encounter for antineoplastic chemotherapy	62,756	177,749	35.3	Encounter for antineoplastic chemotherapy	2,038	177,749	1.1	
Z51.11				Z51.11				
Radiotherapy session	919	8,229	11.2	Age-related cataract	1,061	21,255	5.0	
Z51.0				H25.900				
Systemic lupus erythematosus	607	4,620	13.1	Unspecified disorder of refraction	544	5,134	10.6	
M32.0				H52.7				
Systemic lupus erythematosus with: kidney involvement	296	1,585	18.7	Radiotherapy session	323	28,108	1.1	
M32.1 + N085				Z51.0				
Multiple myeloma	290	616	47.1	Systemic lupus erythematosus	188	4,620	4.1	
C90.0				M32.0				
Unspecified disorder of refraction	214	5,134	4.2	Iridocyclitis	69	7,492	0.9	
H52.701				H20.9				
Age-related cataract	213	21,255	1.0	Chronic obstructive pulmonary disease with acute exacerbation	67	4,457	1.5	
H25.9				J44.1				
Malignant neoplasm of bronchus and lung	202	7,763	2.6	Systemic lupus erythematosus with: kidney involvement	64	1,585	4.0	
C34				M32.1 + N085				
Encounter for other specified aftercare	111	2,773	4.0	Hemiplegia, unspecified	51	2,063	2.5	
Z51.89				G81.9				
Malignant neoplasm of rectum	89	4,588	1.9	Acute pancreatitis	47	9,350	0.5	
C20.0				K85.9				

### Comparison of departments with planned and unplanned readmissions

Planned readmissions were most common in the department of head and neck oncology both in terms of the total number (38,461) and readmission rate (45.0%) followed by the department of hematology (9,851; 29.9%). Head and neck oncology had the highest number of unplanned readmissions (1,172; 1.4%), followed by nephrology (595; 1.5%), followed by the department of nephrology (595, 1.5%) and hematology (547; 1.7%). The highest rate of unplanned readmission was in the department of rheumatology (2.2%), followed by the department of ophthalmology (2.1%) and hematology (1.7%). The number and rate of planned and unplanned discharges in the department of internal medicine were much higher than the number and rate of planned and unplanned admissions in the department of surgery (Table 4).

**Table 4:** Characteristics of hospital departments

	Department	Planned N	Discharged N	(%)	Department	Unplanned N	Discharged N	(%)
Internal medicine	Head & Neck Oncology	38,461	85,464	45.0	Head & Neck Oncology	1,172	85,464	1.4
	Hematology	9,851	32,968	29.9	Nephrology	595	41,130	1.5
	Abdominal Oncology	9,154	57,504	15.9	Hematology	547	32,968	1.7
	Thoracic Oncology	6,214	37,192	16.7	Thoracic Oncology	417	37,192	1.1
	Lung Cancer Center	1,986	16,470	12.1	Rheumatology	416	18,721	2.2
Surgery	Ophthalmology	659	89,155	0.7	Ophthalmology	1,749	84,742	2.1
	Gastrointestinal Surgery	465	39,480	1.2	Urology	247	45,287	0.6
	Urology	158	46,966	0.3	Liver Surgery	223	30,413	0.7
	Orthopedics	84	63,487	0.1	Orthopedics	173	63,487	0.3
	Breast Surgery	73	28,228	0.3	Gastrointestinal Surgery	172	39,480	0.4

### Annual distribution of planned and unplanned readmissions

The number of planned readmissions and planned readmission rates increased progressively with the number of admissions except in 2020. The number of planned readmissions and the readmission rate (15,996; 7.4%) were the highest in 2019. The number and readmission rate of unplanned readmissions (2,451; 1.3%) were highest in 2020 (Table 5).

**Table 5:** Chronological distribution of planned and unplanned readmissions

Year	Discharged N	Planned (N, %)	Unplanned (N %)
2015	163,515	8,461 (5.2%)	1,245 (0.8%)
2016	170,567	10,901 (6.4%)	1,432 (0.8%)
2017	185,499	12,377 (6.7%)	1,352 (0.7%)
2018	201,604	14,845 (7.4%)	1,471 (0.7%)
2019	215,289	15,996 (7.4%)	1,944 (0.9%)
2020	181,963	11,914 (6.5%)	2,451 (1.3%)
Total	1,118,437	74,494 (6.7%)	9,895 (0.9%)

### Monthly distribution of planned and unplanned readmissions

Among the 12 months of the year, the highest number of planned readmissions was recorded in November (7,283; 7.2%), followed by September (7,201; 7.2%) and July (7,108; 7.2%). The highest number of unplanned admissions was recorded in September (1,020; 1.0%), followed by November (1,008; 1.0%) and December (984; 0.9%). The month with the lowest number of both planned and unplanned admissions was February (4,372; 7.0% compared with 435; 0.7%) (Table 6).

**Table 6:** Monthly distribution of planned and unplanned readmissions

Month	Discharged	Planned	Unplanned
Jan	91,861	5,139(5.6%)	713(0.8%)
Feb	62,498	4,372(7.0%)	435(0.7%)
Mar	92,250	5,455(5.9%)	603(0.7%)
Apr	94,257	5,783(6.1%)	736(0.8%)
May	95,082	6,378(6.7%)	787(0.8%)
Jun	95,304	6,263(6.6%)	806(0.8%)
Jul	98,968	7,108(7.2%)	982(1.0%)
Aug	95,855	6,549(6.8%)	868(0.9%)
Sept	99,573	7,201(7.2%)	1,020(1.0%)
Oct	85,846	6,765(7.9%)	953(1.1%)
Nov	101,190	7,283(7.2%)	1,008(1.0%)
Dec	105,753	6,198(5.9%)	984(0.9%)
Total	1,118,437	74,494(6.7%)	9,895(0.9%)

## Discussion

In this study, we analyzed planned and unplanned 30-day readmission rates and associated characteristics at a large general university hospital in China. It is the only hospital in the region with the highest referral rate across all medical specialties and it treats the most complex and difficult cases, which are more likely to be readmitted.

We found some significant differences between 30-day planned and unplanned readmission patients. According to our study, women are twice as likely as men to have plan readmission. However, there were 15.7% points more female patients with planned readmissions than female patients with unplanned readmissions. There were significantly more female patients with planned readmission than those with unplanned readmission (P < 0.001). Some studies identified men as a risk factor for 30-day readmission [13]. However, most studies that included sex-based readmission showed no difference between sex and readmission rate [14, 15]. Although gender may be a risk factor for readmission in some diseases [14], large prospective studies of gender-related readmission are needed.

The proportion of patients living with a spouse was significantly higher for planned readmissions than for unplanned readmissions, and the proportion of divorced, single and widowed patients was significantly higher for unplanned readmissions than for planned readmissions (p < 0.001). This finding is consistent with previous studies showing that marriage has a protective effect on unplanned readmission [16–18].

In this study, the interval from discharge to readmission was mainly concentrated in 20≤-≤30 days for both planned and unplanned readmission patients, 55.9% and 45.7%, respectively, the interval between planned readmission was significantly higher than that of unplanned readmission (18.54±7.96 vs. 16.18±10.01 days). The results of this study showed that 5,683 patients (7.6%) had planned readmission within 7 days (1≤-<7), of which 1,262 (1.7%) were readmitted within 3 days (1 ≤-<3). From a clinical perspective, this may be a misclassification of planned readmissions. This requires additional validation work (review of the medical records) to examine in more detail planned readmissions that may have been misclassified [19]. Most studies suggested that a 7-day cut-off is an effective intervention point for early and preventable readmissions [19]. Readmissions within the first seven days after hospital discharge were more likely to be preventable than those occurring in a late period of 8–30 days [19–21]. Some studies have shown that early readmissions ( ≦ 7 days) within 30 days of discharge are twice as likely to be preventable as late readmissions, with adjusted preventability rates decreasing significantly after day 7 post-discharge. Readmissions within one week of discharge were more likely to be preventable [20, 22, 23]. Other studies considered readmissions that occurred within 0 to 10 days were judged to be preventable [24].

In this study, oncology patients had the highest number of chemotherapy treatments in both planned and unplanned readmissions. This is related to the highest number of oncology patients hospitalized in this study. The number and proportion of planned readmissions in internal medicine were much higher than in surgery. With the exception of ophthalmology, the number and proportion of unplanned readmissions were much higher in internal medicine than in surgery. Planned readmissions were primarily related to the specific nature of a disease, which is considered unavoidable because it results from a typical clinical pathway [25]. However, insight into planned readmissions can facilitate the efficient allocation and optimization of healthcare resources.

Based on the results of this study, there was a correlation between planned readmissions of patients, with the number of hospital admissions increasing each year from 2015 to 2019, as did the number of planned readmissions. A decrease in both inpatient admissions and planned readmissions in 2020 due to the COVID-19 pandemic. However, we found no correlation between the number of unplanned readmissions and hospital admissions. In particular, although the number of hospitalizations was lower in 2020 than in 2017, the number and proportion of unplanned readmissions were the highest. This may be related to COVID-19 affecting the health status of patients or the quality of care. This reason needs to be investigated further.

Throughout the year, planned and unplanned readmissions showed a monthly distribution over the last 6 years. The highest numbers of planned and unplanned readmissions were recorded in November (7,283; 7.2%) and September (1,020; 1.0%). However, the number of planned and unplanned readmissions was lowest in February (4,372; 7.0% vs. 435; 0.7%). February is usually the Chinese Lunar New Year, and due to traditional Chinese culture, hospital visits are not usually made during the New Year [26]. This factor is often specific to the context of Asian countries and reflects the social and cultural context. Therefore, we believe that social and cultural factors are also the influencing factors of planned and unplanned readmission.

### Limitation

There are several limitations to this study. First, the study used data from a university hospital, and our findings may only apply to similar providers, so generalizing these findings to other types of hospitals may be risky. Second, the study was retrospective and included a limited number of variables, so it is subject to residual confounding and may differ from the true causal effect. Third, we validated the ICD-10 codes; there may be inaccuracies in the coding that could introduce imprecision into our estimates.

## Conclusion

This study found that social and cultural factors may also influence planned and unplanned readmissions. Planned readmissions of less than 7 days may be misclassified and should be reviewed as unplanned readmissions. The study of planned readmissions can help to optimize and allocate healthcare resources. Analysis of risk factors for unplanned readmissions (LOS, male, separated/divorced, single, widowed/other, ICU stay and surgery) will help identify key combinations of interventions that are effective in preventing readmissions.

## Data Availability

Data cannot be shared publicly because of West China hospital regulations. Data are available from the Bioethics Committee at West China Hospital, Sichuan University. (contact via 86-28-85422654) for researchers who meet the criteria for access to confidential data.

## Abbreviations

HRRP: Hospital Readmissions Reduction Program
HER: Electronic Health Record
ICD-10: International Classification of Diseases, 10th Revision
